# Amniotic Fluid Stem Cells: Future Perspectives

**DOI:** 10.1155/2012/741810

**Published:** 2012-06-06

**Authors:** Margit Rosner, Katharina Schipany, Bharanidharan Shanmugasundaram, Gert Lubec, Markus Hengstschläger

**Affiliations:** ^1^Institute of Medical Genetics, Medical University of Vienna, Währinger Strasse 10, 1090 Vienna, Austria; ^2^Department of Pediatrics, Medical University of Vienna, Währinger Gürtel 18-20, 1090 Vienna, Austria

## Abstract

The existence of stem cells in human amniotic fluid was reported for the first time almost ten years ago. Since this discovery, the knowledge about these cells has increased dramatically. Today, amniotic fluid stem (AFS) cells are widely accepted as a new powerful tool for basic research as well as for the establishment of new stem-cell-based therapy concepts. It is possible to generate monoclonal genomically stable AFS cell lines harboring high proliferative potential without raising ethical issues. Many different groups have demonstrated that AFS cells can be differentiated into all three germ layer lineages, what is of relevance for both, the scientific and therapeutical usage of these cells. Of special importance for the latter is the fact that AFS cells are less tumorigenic than other pluripotent stem cell types. In this paper, we have summarized the current knowledge about this relatively young scientific field. Furthermore, we discuss the relevant future perspectives of this promising area of stem cell research focusing on the next important questions, which need to be answered.

## 1. Introduction

Although human amniotic fluid cells are widely used in routine prenatal diagnosis, the knowledge about these cells remains limited. However, the notion that undifferentiated and differentiated cells of varying origins and lineages are present in amniotic fluid has been supported by several reports over the last three decades. This is not surprising, considering that cells belonging to the amniotic epithelium, fetal skin, and the fetal urogenital, respiratory, and gastrointestinal systems have been detected in amniotic fluid. During prolonged gestation, fetal respiratory, urine, and gut secretions can be found in the amniotic fluid. In addition, it is also known that the composition, the morphology, and the growth properties of amniotic-fluid cell samples are affected by certain fetal pathologies, such as for example, neural tube defects or gastroschisis [1–3].

New interest in amniotic fluid-derived cells was initiated by two independent findings. In 2001, it was suggested that amniotic fluid cells could be used in tissue engineering approaches for the surgical repair of congenital anomalies in the perinatal period. The authors mechanically isolated a subpopulation of cells from amniotic fluid of pregnant ewes with a distinct morphology. The immunocytochemical profile of these cells was very comparable to that of cells of a mesenchymal, fibroblast/myofibroblast lineage. Exhibiting significantly faster proliferation than comparable fetal and adult cells, these amniotic fluid-derived cells could be cultivated on polyglycolic acid polymer scaffolds up to confluent cell layers [4]. It has originally been discussed that such an engineered construct would be optimal to function as a graft for implantation either in the neonatal period or even before birth. This could be of special interest for children born with a body wall defect, who are too young for a graft to be taken from elsewhere in their bodies for reconstructive surgery [4, 5]. The results obtained in animal models are indeed encouraging. However, to the best of our knowledge, we are not aware of a report describing the clinical use of such a cell-based therapy approach in humans until now.

 Another finding on amniotic fluid cells initiated a very promising and rapidly growing research field. Almost ten years ago, the first suggestion of human amniotic fluid as a new putative source for stem cells was published [5–7]. The first evidence for the existence of AFS cells was demonstrated by the discovery of a highly proliferative cell type in human amniotic fluid expressing the pluripotent stem cell marker Oct4. Beside the fact that these cells express markers known to be specific for pluripotent stem cells, they were proven to express cell cycle proteins known to be specific for cycling cells [5–8]. After this first description, many groups have confirmed the existence of these Oct4+/c-Kit+ AFS cells and have reported their potential to differentiate into hematopoietic, neurogenic, osteogenic, chondrogenic, adipogenic, renal, hepatic, and various other lineages [9–19]. Although, regarding their biological properties and marker expression pattern, AFS cells appear to be more similar to embryonic stem (ES) cells than, for example, to trophoblast cells, the precise origin of AFS cells remains elusive. Biochemical, immunocytochemical, biological, and morphological investigations revealed that AFS cells represent a new and specific entity, being distinct from ES cells or other stem cell types, such as the ones which can be isolated from amniotic epithelial or trophoblastic sources. Today it is of great interest to clarify two relevant questions with regard to AFS cells. Where do they come from? Do they have an *in vivo* biological function? We have already earlier discussed that AFS cells could probably play a role in intrauterine wound healing processes. However, so far there exists no experimental support for this hypothesis. It is obvious that experimental settings allowing to prove this hypothesis are not really easy to imagine or practical at this time (or yet to be developed) [9–19].

Since their first discovery, it was of highest importance to clarify the question whether AFS cells really harbour pluripotent differentiation potential via successfully initiating the differentiation into different lineages starting from one single stem cell. It is relevant to note that many different reports in the literature claiming to describe research on AFS cells did not even clarify what kind of cells they are working with. Quite often, the investigators just used a mixture of cells from amniotic fluid obtained via specific cultivation procedures. However, as mentioned above, such amniotic-fluid-derived cell mixtures contain a wide variety of specific undifferentiated and differentiated cell types. Whenever a study reports a differentiation potential upon specific cell lineages, it is of highest relevance to first clarify which starting cell type was used (by detailed biological and immunocytochemical characterization). Furthermore, the proof that AFS cells really harbour pluripotent differentiation potential can only be obtained starting with one single cell characterized to be a stem cell. In any other case, one could assume that a mixture of amniotic fluid cells, which has been used as starting material, very likely contained a cell type with the potential to differentiate into a specific lineage and other cell types with other differentiation potentials. Or the used *in vitro* differentiation protocol used in some studies induced a selection (via a growth advantage) for an already (included) differentiated cell type rather than the bona fide differentiation. Single cell approaches are obligatory and practical after minimal dilution experiments.

The first research group, which was really taking that into account, reported that descending from one single Oct4-positive AFS cell, it was possible to induce adipogenic, osteogenic, and neurogenic differentiation [10]. The authors used a two-stage culture protocol followed by a detailed immunocytochemical characterization of the obtained stem cell type [10]. Three years later, another research group isolated monoclonal AFS cells via flow cytometric selection and minimal dilution, which expressed the stem cell markers c-Kit and Oct4 [14]. The authors described the first establishment of monoclonal AFS cell lines, harbouring a high proliferative potential, which could be cultivated for many cycling periods with a stable chromosomal status. Using such AFS cell lines allowed them to demonstrate that adipogenic, osteogenic, myogenic, endothelial, neurogenic and hepatic cell differentiation could be induced. Importantly, these authors also reported that AFS cells, unlike ES cells, do not induce tumor formation in severe combined immunodeficient (SCID) mice (for a detailed discussion of this aspect see below) [14].

ES cells, when cultivated in the absence of differentiation factors, can spontaneously form three-dimensional multicellular aggregates called embryoid bodies. In the past, embryoid bodies have been widely considered as an optimal starting point for the differentiation of stem cells into various lineages. Accordingly, embryoid body formation followed by different differentiation-inducing approaches is seen as an appropriate way to prove the pluripotent differentiation potential of a specific stem cell type [8, 20]. Consequently, it was of interest to test whether, starting from one single cell, AFS cells are capable of forming embryoid bodies. Indeed, monoclonal human AFS cells can form embryoid bodies, when cultured without antidifferentiation factors under conditions in which they are unable to attach to the surface of culture dishes and without contact to feeder cells. The formation of such three-dimensional multicellular aggregates is accompanied by a decrease of stem cell marker expression and by the induction of differentiation into different lineages [20]. This study demonstrating the potential to form embryoid bodies was the ultimative proof of AFS cells to be pluripotent. In addition, it now allows the recapitulation and investigation of the three-dimensional structures and tissue-level contexts of many differentiation phenomena during early mammalian embryogenesis [20]. These findings on the pluripotency of AFS cells were obtained using monoclonal cell lines generated via magnetic cell sorting and minimal dilution approaches from human amniocentesis samples. Today, many different established monoclonal lines exist, which can be expanded as immature stem cells with high proliferation rate in culture without the need of feeder cells [14, 20, 21].

Taken together, the current status of knowledge is that AFS cells harbour the potential to differentiate into cell types of the three germ layers (ectoderm, mesoderm, and endoderm) and can form embryoid bodies, known as the principal step in the differentiation of pluripotent stem cells. Compared to other types of stem cells, such as adult stem cells, ES cells, or induced pluripotent stem (iPS) cells, AFS cells, have specific advantages. Adult stem cells are often hard to sample, exhibit lower differentiation potential than AFS cells and cannot be grown with high proliferative activity. The generation of ES cell lines via destroying a human embryo raises a variety of ethical issues, which are discussed differently from country to country. Furthermore, ES cells are tumorigenic, whereas AFS cells, as already mentioned above, do not induce tumor formation in severe combined immunodeficient mice. Compared to iPS cells, there is no need for ectopic induction of pluripotency in AFS cells. AFS cells are genomically stable and do neither harbour the epigenetic memory nor somatic mutations of already differentiated source cells. Furthermore iPS cells have been reported to accumulate karyotypic abnormalities and gene mutations during propagation in culture. Recently, it has been reported that during the ectopic induction of pluripotency iPS cells only incompletely recapitulate their epigenetic pattern. This important finding must be taken into account when these cells are planned to be used for detailed investigations on differentiation processes as well as when they are considered for new putative therapeutic approaches. AFS cells already exhibit stem cell properties and do not need ectopic induction of pluripotency. Furthermore, AFS cells already exhibit the epigenetic pattern of stem cells. In summary, it is not surprising that many attempts are currently focusing on the question under which conditions AFS cells could be used for stem-cell-based therapies. Furthermore, AFS cells are currently becoming increasingly accepted as an optimal tool for basic research [3, 8, 22–24].

 Although ES cells, iPS cells, and AFS cells are considered to harbour a pluripotent differentiation potential, the question of whether they exhibit the same qualitative spectrum of differentiation potential remains unanswered. Pluripotent stem cells are defined as self-replicating cells (the cells can divide *per se*) known to have the capacity to develop into cells and tissues of the primary germ layers, ectoderm, mesoderm, and endoderm. These three stem cell types (ES, iPS, and AFS cells) have been demonstrated to harbour the potential to differentiate into cells of the three germ layers. All three can also form embryoid bodies. However, whether they really have comparable potentials to differentiate into a specific cell type with all its known biological functions must be tested from case to case. In fact, we believe that it is necessary to directly examine and compare their differentiation potentials and select the most suitable cell types for basic science projects and for the putative usage in new stem-cell-based therapies. Furthermore, one obvious difference between these three pluripotent stem cell types should be investigated in more detail in future. Since the first description of their *in vitro* cultivation, ES cells have been known to be tumorigenic. Similarly, iPS cells induce tumor formation, when they are subcutaneously transplanted into nude mice. However, AFS cells have been reported not to form tumors in severe combined immunodeficient mice. Since the latter has so far only been studied in one project analysing a specific set of animal transplantations, further investigations are warranted to clarify whether AFS cells are really not tumorigenic. Obviously, if it holds true, this would be an important advantage over ES and iPS cells at least with respect to a putative clinical usage [3, 8, 14, 19, 22–25].

## 2. AFS Cells for Therapy: Future Perspectives

Much of the excitement surrounding human stem cells is connected with the hope of clinicians and patients that these cells can once be used for cell therapies for a wide spectrum of human diseases. Here it must clearly be stated that the work on AFS cell-based therapies is still in its infancy. Many questions are currently under investigation, and so far no therapeutic approach based on AFS cells has reached the level of clinical routine application. However, a variety of new research results provide strong evidence that AFS cells could indeed serve a powerful tool in regenerative medicine [3, 8, 24, 25].

For example, acute and chronic renal failures are disorders with high rates of morbidity and mortality. Kidney transplantation remains the most effective treatment option for a majority of patients with end-stage renal disease. Unfortunately, shortage of compatible organs is a very limiting factor. Treatment strategies are also based upon conventional renal dialysis, but the mortality rate of patients requiring chronic dialysis is high. Accordingly, the putative usage of stem cells in the repair of kidney injury came into focus. Several recently published studies on renal differentiation of AFS cells make it tempting to speculate that these stem cells could once be considered as a new promising source for cell-based therapies to repair kidney injury and warrant further investigations into this direction [24, 26–30]. Using a kidney reaggregation assay, we have recently published that AFS cells harbour the potential to differentiate upon nephrogenic lineages and that this capacity depends on the mammalian target of rapamycin (mTOR) signalling pathway [28] (see also the discussion below). Others have demonstrated that human AFS cells can integrate into renal tissues when injected into isolated murine embryonic kidneys [27] or that injection of AFS cells into damaged kidneys of mice with rhabdomyolysis-related acute tubular necrosis can mediate a protective effect [29]. Although these and other data make it tempting to speculate that AFS cells may provide successful alternative approaches for the treatment of, for example, acute tubular necrosis, many more questions must be answered before such cell-based therapies can be considered for routine applications in humans.

For many different reasons the establishment of new stem-cell-based therapies for heretofore incurable central nervous system pathologies, such as Parkinson's disease, spinal cord injury, multiple sclerosis, or stroke, is also of great interest. Neural stem cells, which have been investigated for this purpose, can be found in the adult central nervous system and in the developing embryo, but these tissues are not easily available and raise ethical concerns. In the recent years, different groups have reported on the neurogenic differentiation potential of AFS cells. However, before the next steps into the direction of the clinical usage of AFS cell-based approaches can be considered, the proof that AFS really can form mature neurons must be provided. In fact, there is still an ongoing debate in the literature, discussing whether AFS cells are really able to form functional neurons. In the near future, it will be very important to find out what kind of neurogenic cell types can be developed from AFS cells. The question whether AFS cells can differentiate into functional mature neurons must be investigated by analysing the ability to fire tetrodotoxin-sensitive action potentials with the characteristic shape and duration or by demonstrating synaptic communication by electron microscopy [10, 13–16, 18, 19, 31].

Here, it would be possible to discuss some more examples for putative therapeutic approaches using AFS cells. Sometimes it is argued that many basic questions regarding the origin, the tumorigenicity, the differentiation potential, the epigenetic status, or the genomic stability must be investigated before AFS cells could further be considered as a therapeutic tool. However, we believe all these aspects should be studied in parallel. In addition, for future considerations it is really important to quantitatively and qualitatively compare all these properties of AFS cells to those of other pluripotent or adult stem cell types.

## 3. AFS Cells in Basic Science: Future Perspectives

Stem cells are very useful tools to study the molecular and cellular regulation of differentiation processes. One approach to learn more about the role of, for example, a specific gene for a certain differentiation process is to knock down the endogenous expression of the gene of interest. Such an approach allows to clarify the role of modulated gene expression for the cell potential to differentiate into a specific lineage. We recently published a protocol for efficient siRNA-mediated prolonged gene silencing in AFS cells [21]. This protocol, which we already tested for a variety of different genes, allows a 96–98% downregulation of the endogenous gene expression over a time period of about 14 days in AFS cells and in a variety of other primary, immortalized, or transformed cells [21].

More recently, we have made use of this approach to study the role of the mTOR pathway in human AFS cells. Deregulation of upstream regulators of mTOR, such as, for example, Wnt, Ras, TNF-*α*, PI3K, or Akt, is a hallmark in many human cancers. Mutations in the mTOR pathway component genes *TSC1, TSC2, LKB1, PTEN, VHL, NF1,* and *PKD1* trigger the development of the human genetic syndromes: tuberous sclerosis, the Peutz-Jeghers syndrome, the Cowden syndrome, the Bannayan-Riley-Ruvalcaba syndrome, the Lhermitte-Duclos disease, the Proteus syndrome, the von Hippel-Lindau disease, neurofibromatosis type 1, and polycystic kidney disease. Beside a variety of single-gene disorders and tumorigenesis, the mTOR pathway has also been shown to be of relevance for the development of complex diseases, such as cardiac hypertrophy, obesity, or type 2 diabetes. All these pathological consequences of deregulated mTOR activity are explainable, considering that mTOR is the key component of the insulin signalling cascade, which is involved in a wide variety of different processes such as cell growth, proliferation, metabolism, transcription, translation, survival, autophagy, aging, differentiation, and oncogenesis [8, 20, 21, 28, 32]. We found that the entire process of embryoid body formation of AFS cells depends on both mTOR-containing enzymes, mTORC1 and mTORC2 [20]. As mentioned above, modulating mTOR components via specific siRNA approaches revealed that the potential of AFS cells to contribute to renal tissue formation is regulated by this signalling pathway [28]. More recently, the approach to knock-down endogenous gene functions in AFS cells allowed us to detect that the two mTOR regulators, tuberin and PRAS40, are antiapoptotic gatekeepers during early human AFS cell differentiation [32]. Taken together, we strongly believe the approach of siRNA-mediated knockdown of endogenous gene expression in monoclonal human AFS cell lines to be a very powerful tool for future projects dealing with the molecular regulation of differentiation [3, 8].

Another very interesting aspect for future basic research is the banking of AFS cell lines carrying naturally occurring mutations, which are of relevance for certain human pathological phenotypes. In medical genetics the future development of new prophylactic and therapeutic strategies directly depends on a better understanding of the mechanisms by which naturally occurring genetic variation contributes to disease [33]. In countries, where it is legal to use human embryos for research, ES cell lines carrying certain inherited defects are generated from embryos with all kinds of numerical chromosomal abnormalities or specific monogenic disease mutations excluded from transfer into the uterus after preimplantation genetic diagnosis [34]. Also a variety of iPS lines from single-gene disorders, chromosome syndromes, and complex diseases have already been generated, with the aim to use them for basic research projects [35]. Still, as already discussed in detail, both ES cells and iPS cells harbour relevant disadvantages compared to AFS cells. Beside other invasive approaches, amniocentesis is a widely accepted standard procedure of prenatal care since the 1970s. It is almost unpredictable how many amniocenteses are worldwide performed per year. Taken together, we believe that generation and banking of normal human AFS cell lines and of AFS cell lines with chromosomal aberrations, as well as of AFS cell lines with specific monogenic disease mutations could provide very powerful tools for disease modelling in future research. Here it is important to note that banking of AFS cells for non-research purposes, with the aim to protect a child's health by having stem cells available throughout his or her lifetime, is something else. Some companies in Europe and the USA are already offering to bank AFS cells, when, for example, an amniocentesis is performed for prenatal diagnosis. Their arguments for the preservation of AFS cells are that these cells once could help treating injuries (e.g., repairing cartilage for the knee), healing wounds, or developing skin for specific grafts. As mentioned earlier, in future, extensive research is required to establish the putative clinical use of AFS stem cells in humans. The promising results obtained during the last few years within this still young scientific field clearly warrant further detailed investigations into the direction of putative clinical application of AFS cells. In this paper we would like to emphasize that banking of AFS cells with natural occurring mutations for human genetic research should have started as soon as possible in different laboratories under comparable high-quality standards. It would be worth to encourage different laboratories to sample amniotic fluid from amniocentesis with comparable indications from similar weeks of pregnancy. The protocols to isolate stem cells, perform minimal dilutions, and characterize the so-obtained monoclonal AFS cell lines should be standardized. Biobanking of AFS cell lines with characterized mutations would allow to jump to the next step of human genetic research using human stem cells [3, 8]. 
